# Network effects in environmental justice struggles: An investigation of conflicts between mining companies and civil society organizations from a network perspective

**DOI:** 10.1371/journal.pone.0180494

**Published:** 2017-07-07

**Authors:** Cem Iskender Aydin, Begum Ozkaynak, Beatriz Rodríguez-Labajos, Taylan Yenilmez

**Affiliations:** 1 REEDS-Centre for Research in Ecological Economics, Eco-Innovation and Tool Development for Sustainability, UVSQ-Universite Paris Saclay, Guyancourt, France; 2 Department of Economics, Bogazici University, Istanbul, Turkey; 3 Institute of Environmental Science and Technology (ICTA), Autonomous University of Barcelona, Barcelona, Spain; 4 Independent scholar, Istanbul, Turkey; Universidad Rey Juan Carlos, SPAIN

## Abstract

This paper examines conflicts that occur between mining companies and civil society organizations (CSOs) around the world and offers an innovative analysis of mining conflicts from a social network perspective. The analysis showed that, as the number of CSOs involved in a conflict increased, its outcome was more likely to be perceived as a success in terms of environmental justice (EJ); if a CSO was connected to other central CSOs, the average perception of EJ success was likely to increase; and as network distance between two conflicts increased (or decreased), they were more likely to lead to different (or similar) EJ outcomes. Such network effects in mining conflicts have policy implications for EJ movements. It would be a strategic move on the part of successful CSOs to become involved in other major conflicts and disseminate information about how they achieved greater EJ success.

## 1. Introduction

The mining industry enjoys a prominent role in today’s global economy. Growth in consumption and production has escalated the need for energy and raw materials, with resource use reaching exceptionally high levels worldwide [1–3] leading to a concomitant rise in the number of conflicts over resource extraction and waste disposal. Mining activities generate various negative environmental and social impacts, including deforestation, biodiversity loss, high water consumption, groundwater contamination and population migration [4–6]. These all create discontent and conflict because the basic needs of some groups and their access to environmental resources and services are compromised, resulting in the loss of livelihoods, cultures, and even lives [7–9].

There is an extensive and interdisciplinary body of scholarly work that utilizes qualitative and quantitative social research methods to analyze mining activities and conflicts from spatial, socio-economic and ecological distribution perspectives [10–13]. The environmental justice (EJ) and political ecology literatures, for instance, abound with studies that employ the case study approach to uncover and shed light on the central concerns and claims of the social actors involved in mining resistance struggles [14–21]. Accordingly, civil society organizations (CSOs) active in mining conflicts focus on two issues in particular: on the link between distributional concerns (the need for environmental health and security) and claims for recognition (the defense of basic human rights and indigenous territorial rights) [22–23], and on the importance of participation in decision-making, so as to be able to introduce alternative visions of development in decision-making processes (participation) [10,24]. Consequently, mining conflicts are extremely relevant to the global EJ movement [25].

Moreover, mining conflicts usually occur between local communities/CSOs and multinational/national mining companies, and hence, are widely considered an example of contentious politics. As a result, the literature on collective action and social movements, is both insightful and relevant to better understanding the new wave of mobilization against extractive industries [26], with extensive debates ranging from how CSOs should be structured in their attempts to maximize the strength of collective challenges [27], and their degree of institutionalization [28], to funding sources and the extent of their membership base [29]. Given that institutionalization and professionalization require greater effort to generate financial resources, highly professionalized and institutionalized organizations are criticized, for instance, if they lose touch with the communities they seek to represent.

Whether CSOs should organize and mobilize at the local, national and transnational levels is then another point of discussion that has implications for organizational structure, level of protest, grassroots support, and the overall effectiveness of the mobilization [30–32]. Nationwide organizations, for instance, are said to offer a relatively higher chance of engaging policy-makers and transnational networks, but their grassroots links are often quite weak. Conversely, local organizations are generally celebrated for their grassroots support [33]. There is, in fact, a particular strand of research on social conflict and mobilization which looks at the interaction among political processes, mobilizing structures and framing efforts as a means to explain the emergence and outcomes of social movements looking at the interaction of [34,35]. In the literature, social movements are also regarded as informal relationship networks among diverse organizations that share a distinctive collective identity and mobilize resources [36–38]. As a method, network analysis had already been used to examine numerous real-life situations, including the spread of epidemic diseases, traffic congestions, scientific collaborations, labor market outcomes, ownership structure of global markets and international trade relations[39–45]. After the Great Recession of 2008, for instance, network analysis was used extensively to understand how the financial crisis spread and how connections in financial systems might lead to systemic crisis [46–49]. According to Della Porta and Diani [50], the spread of global justice mobilizations has recently made the role of networks particularly visible in the literature on social movements: examples include coalitions that involve transnational actors and networks as well as local actors on issues such as environmental protection or human rights. Here, it is generally argued that network links make the organization in a movement better equipped to deal with the emergencies and threats in their environment. The theory also suggests that having a significant number of allies increases the chances of success for mobilizing groups [51,52]. No doubt, a network allows—to some extent—mediation between the participatory character of grassroots movements and the coordination guaranteed by formal structures. For example, Malinick et al. [53] found that being centrality positioned in the communication network of environmentalists in British Columbia had a positive impact on the amount of media coverage.

Yet, even though alliance building seems a generally sensible and desirable option, as Della Porta and Diani [50] once again underline, inter-organizational relationships in practice vary markedly in terms of intensity of engagement and nature of organization. While one group of network studies tries to explain differential participation in social movements by looking at network formations [54,55], for instance, another group focuses on network organization models, noting that in contrast to conventional formal organizations, which are based on the vertical integration of multiple units, newly emerging models coordinate activities based on the independence of single components and multiple levels of interaction [38,56]. There are also hybrid models of network organization that develop by combining elements of formality with a loose network structure. A wave of scholarship that emerged on the social movement outcomes in the late 1990s has been influential in improving our knowledge of the conditions under which collective mobilizations produce certain effects [51,57]. Nonetheless, the discussion on “Do networks really matter? And in what forms?” still needs to be bolstered by empirical analysis that employ new data and methods. To date, relatively little has been written systematically on network effects in environmental justice struggles; moreover, the few existing studies underlying the importance of social movement networks do not use the tools of network analysis (see, for instance, 33,56,58).

At this junction, this paper brings together two rich insightful strands in the literature—one focusing on the efforts to better understand what constitutes EJ and transformations toward it, and the other on social movements and outcomes from a network perspective. Based on a collaborative effort that brings together information on mining conflicts and drawing on the tools of social network analysis, it also helps us position mining conflicts and EJ struggles within this growing literature on social movements and networks.

As the first step in the study, we described the nature of the relationships among the corporations involved in mining activities and among the CSOs that stood up to these activities. To this end, the names of the mining companies and CSOs involved in 346 mining conflicts were obtained from The *Atlas of Environmental Justice* (EJAtlas, see https://ejatlas.org)—a database enriched by the interactive discussions of activists and experts as part of the EJOLT (The Environmental Justice Organizations, Liabilities, and Trade) project that ran from 2011 to 2015. [59].

The reporting by activists and CSOs on EJ outcomes then formed the basis of our analyses, which revealed that conflicts were more likely to achieve greater EJ success as the number of CSOs increased, and that CSOs connected to other central CSOs in a network were more likely to achieve greater EJ success on average. More importantly, however, we examined whether network effects were a factor in mining conflicts in terms of EJ success. Analyses revealed that the closer (or more distant) two conflicts were in a network, the more likely they were to have similar (or different) EJ outcomes. Such network effects in mining conflicts have policy implications for EJ movements as it offer insights into the bridges that can be built to improve social movement outcomes. CSOs involved in mining conflicts are continuously looking for better ways to do their job and interpreting their struggles for the masses. As a strategy, CSOs that achieve success should become involved in other major conflicts and share information on how they achieved these results. Overall, the present study builds on recent debates on the importance and ways of coordinating action to make certain themes more pertinent to the political agenda. As Della Porta and Diani [50] indicate intense exchanges between organisations with sufficient interests and motives can produce non-competitive cooperation and collaborate to activate successful joint mobilizations.

The approach adopted here can be seen as a first step in showing how mining conflict databases can be a learning source for activists and how social network analysis can be used as an effective tool to support EJ movements and inform relevant policy questions. To the best of our knowledge, this paper is the first to consider environmental conflicts as a real-life network and proves network effects in a worldwide sample of mining conflicts. While we did not look at network formations per se, the overall aim was to underline the importance of building social movement networks and enhance the capacity of EJ groups. With this paper, it will be possible to shape movement mobilization strategies by taking network structure and network effects into account.

Following this introduction, the data and methods we used are explained in Section 2. Section 3 offers an overall picture of what CSOs face in mining conflicts, network analyses of both the mining companies and the CSOs involved in the reported conflicts, and information on how CSOs stand against mining. Section 4 looks at network effects through an analytical examination of how EJ is perceived in the reported cases of resistance to mining. The final section summarizes the insights gained and outlines various policy recommendations.

## 2. Data and methods

As previously mentioned, the data used in this study was retrieved from the EJAtlas, where systematic information on ecological distribution conflicts are compiled; the information is provided by academics, civil society groups, and individuals who are interested in supporting the efforts of EJ movements. Data collection was a cooperative process that lasted four years: from April 2011, when the project began, to October 2014. The data became public when the map was released in March 2014. Of the 1,200 cases registered at the time, 346 were classified as mining conflicts, and an operational definition was established to select the appropriate entries: Conflicts related to the extraction, processing, and transport of minerals as well as to waste management in *specific* mining projects were defined as mining conflicts. This binds the idea of mining conflicts to localized processes, typically at the local or regional level.

Several levels of data refinement were implemented while information was being gathered. As the cases in the EJAtlas were being compiled, a standard form was sent to collect qualitative and quantitative data for cases reported by CSOs. This helped in identifying what counts as an environmental conflict and in standardizing certain types of information, which made it easier to compare cases. Next, information quality was assured via the systematic moderation of data inputs. Finally, external reviews were sought not only to check the adopted operational definition of mining conflict, but also to avoid as much as possible problems of over/under-representation in filtering the mining cases from the EJAtlas database. Of the 346 mining conflicts identified, the vast majority were related to precious metals (39.0%) such as gold and silver, and base metals (35.8%) such as copper. These were followed by energy-related materials (18.5%) such as coal and uranium and construction-related materials (6.6%) such as sand and limestone. Almost 85 percent of conflicts occurred in rural and semi-urban areas.

In the original EJAtlas database, respondents were asked whether they considered a particular case a success in terms of EJ and to explain their rationale, in an effort to understand how resistance movements define success and failure in the context of a mining conflict. This is a delicate point in that it combines concrete facts with the perception of a resistance movement by activists and the communities they support. Not surprisingly, the justifications that respondents provided varied enormously; this was especially noticeable in cases where the answer was “I'm not sure.” The answers were then classified as “favorable” (such as when a project was halted, compensations were obtained, or the social fabric was strengthened) or “unfavorable” (such as when a project was still operational, failed to comply with legislation, or could potentially be reactivated) from an EJ perspective. The most frequent answer to the EJ question was “No” for almost half of the registered mining conflicts (46%). Indeed, in 35 percent of these cases, not even a single favorable reason was reported in terms of EJ success; these cases were unambiguously recorded as EJ failures and coded with a score of 0, and the remaining 11 percent (that included some favorable reasons) was coded with a score of 1. Among the “Not sure” responses (33% percent), the most common justification (28%) was that the mining project was still operational and the conflict was ongoing, while in the remaining 5 percent, the project had stopped but uncertainty remained about what would happen in the long term. Finally, about 21 percent of the reported mining conflicts were considered an EJ success, although exclusively favorable reasons were provided in only 13 percent of the cases (coded with a score of 5).Then, using this additional qualitative information, the “Yes”, “Not sure” and “No” answers to the EJ perception question were coded on a scale of 0 (complete failure) to 5 (complete success) in an ordinal gradient of “EJ achievements” (see S1 File)

Next, the 600 companies involved in the 346 mining cases being examined were identified from responses to an open-ended question, and used to form a coalition network. Similarly, another coalition network was formed with the 1,092 CSOs and other supporting organizations that had also been identified via an open-ended question (see S1 File). The information on CSOs was then re-coded and grouped under two main categories: (1) *organization type* (community organizations, non-environmental NGOs, environmental NGOs, religious organizations/charities, governmental organizations, human rights organizations, political parties, and research organizations) and (2) *scale of operation* (local, national, international) (see S1 File).

To understand and visualize the network formations (of both the mining companies and the CSOs), we used Gephi^®^, an open-source network exploration and manipulation software. Due to privacy concerns and in an effort to protect various parties from potential threats and attacks, the names of all nodes were kept anonymous. We first constructed a network comprising CSOs and companies, where two nodes were connected if both were jointly involved in a mining conflict. By using ordinary least squares and ordered probit regressions, we found that CSOs with higher PageRank centrality had significantly higher average EJ scores. Second, we constructed a network of mining conflicts to see if outcomes were spread via the connections that CSOs and companies had; we assumed that two conflicts were linked if they had at least one CSO (or company) in common. Ordinary least squares and ordered probit regression results showed that the closer two conflicts were in the network, the more likely it was for them to have similar EJ outcomes. It is also worth noting that our findings are very robust because we controlled not only for the interaction of country characteristics of the two conflicts, but for conflict fixed effects as well.

## 3. The mining conflict networks: What do EJOs face? How do they react?

This section provides an overview of the social resistance against mining. It does so by revealing the main parties involved in mining conflicts, thereby offering a broader understanding of the conditions CSOs and mining companies encounter when they are involved in these conflicts. In this context, first the relationships and coalitions among the 600 national and international mining corporations involved in the 346 reported cases were thoroughly examined. 79 were identified as subsidiaries of other bigger companies and thus merged with them, resulting in a total of 521 companies (see S1 File). In the network of companies, a company pair was connected if both companies took part in the same project that gave rise to a conflict. Next, a network of CSOs was built on the basis of the 1,092 groups that mobilized against mining projects. Again, similar to the network of companies, 23 NGOs were identified as the national branches of big international NGOs, and hence are merged with their international headquarters (see S1 File), resulting in a total of 1069 groups. In the network of CSOs, two CSOs were connected if both parties were involved in the same conflict.

**Fig 1:**
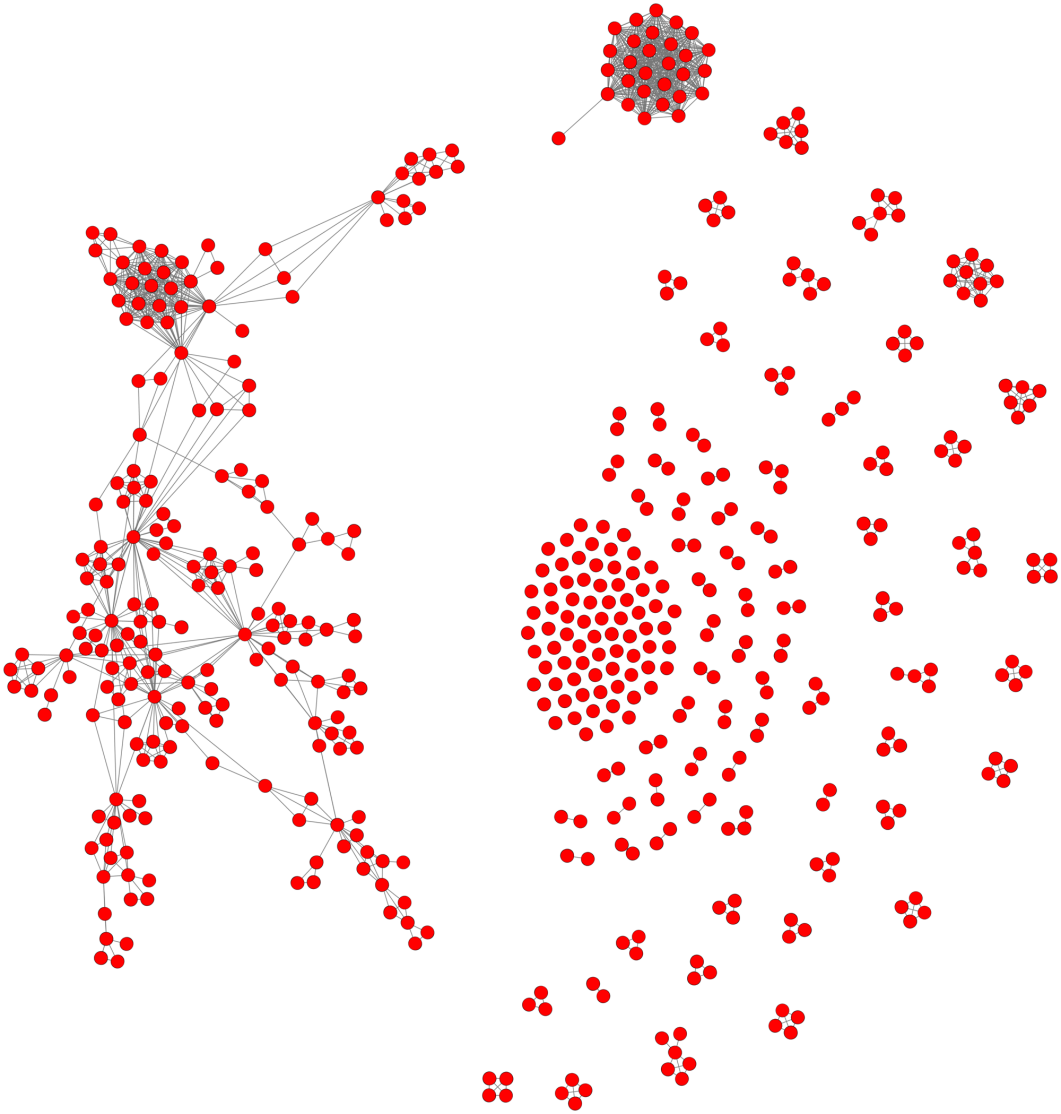
The network of mining companies - The GSCC on the left. (Note: Red nodes depict companies; blue links depict company-to-company links).

Analyses revealed that the network of mining corporations consisted of many different-sized components (sub-networks). A detailed view of this network is provided in Fig 1, which shows that almost half of the conflicts were located in the so-called giant strongly connected component (GSCC)—the main sub-network where nodes are highly interconnected. The GSCC contained 202 companies (40% of the companies); it had a total of 614 links, with an average of 3.03 links per company.

In the GSCC, most of the companies central to the network (i.e. involved in many conflicts) were well-known international companies, with headquarters based in Australia, Brazil, Canada, South Africa, Switzerland and the U.K. They were well connected not only among themselves, but also to other national firms. Many multinationals in the network also had their own national subsidiaries. CSOs are of the opinion that this is a strategy to either overcome national regulations that prevent the participation of international investors or deliberately hide the involvement of multinational companies [60]. Another important point is that not all companies were specialized in mining; some were commodity traders; a finding that underlines the important role international trade plays as a driving force in local conflicts.

Moreover, not all important or well-known companies were in the primary component of the network. Some were weakly connected to the GSCC or simply located in smaller and more isolated (but not less important) components of the network. These companies have their own spheres of influence for a particular commodity or in a specific region. A uranium or base metal mining giant, for instance, may have its own small mining network and create its own sphere of influence by forming coalitions with its local subsidiaries. As a result, in some cases, a company that relatively unknown by the public can be involved in conflicts that are geographically far apart; e.g., one in Turkey and one in Mexico.

The fact that these companies were collectively observed in a network does not mean they all follow the same policies in terms of how they respond to anti-mining protests or relate to communities that oppose mining. However, demonstrating that a network of relationships exists among companies with regard to conflict brings two aspects to the table. First, mining companies have a common, albeit differentiated, interest in responding to mining conflicts, which arguably creates difficulties for their business operations [61]. Second, should a common framework to tackle conflicts be established, a network of corporate relationships would facilitate its development, dissemination, and operation. The Global Mining Initiative, for instance, promoted by the International Council on Mining and Metals (ICMM), is an example of a globally-shared discourse that uses “sustainable mining” as a slogan and presents the industry as a generator of societal benefits while legitimizing access to resources and intervention in the social life of communities and regions No doubt, analyses of mining companies is useful for CSOs as well, especially in determining where their resistance movements fit within the broader picture and showing where it might be beneficial to collaborate and join forces.

Looking at the groups that mobilize against mining in terms of organization type, it was found that of the 1,069 entities named, environmental CSOs were represented the most (42.4%), followed by non-environmental CSOs (27.8%) and community organizations (18.9%) (Table 1). Research organizations (4.1%), human rights organizations (2.0%), religious organizations (2.7%) and political parties (1.5%) also had some presence in the data set. In seven instances, it was governmental organizations that were fighting for EJ. In 21 of the 346 cases, no particular EJO or other organized group came to the fore; in these cases, local residents were the main resistance group.

**Table 1:** Mobilized organizations according to type.

Organization type	Node Color	Frequency	Percentage
Environmental NGOs	Green	453	42.4
Non-environmental NGOs	Dark blue	297	27.8
Communities/Residents	Red	202	18.9
Research organizations	Orange	44	4.1
Religious organizations/Charities	Yellow	29	2.7
Human rights organizations	Purple	21	2.0
Political parties	Light blue	16	1.5
Governmental organizations	Brown	7	0.7
Total # of organizations		1,069	

Source: Authors’ calculations based on data from the EJAtlas

It is also worth noting that 189 of the total number of reported entities (17.3%) were already networks themselves (e.g., platforms, alliances, campaigns, coalitions, and movements). This suggests that anti-mining activists are well aware of the value of cooperation and collaboration.

**Fig 2:**
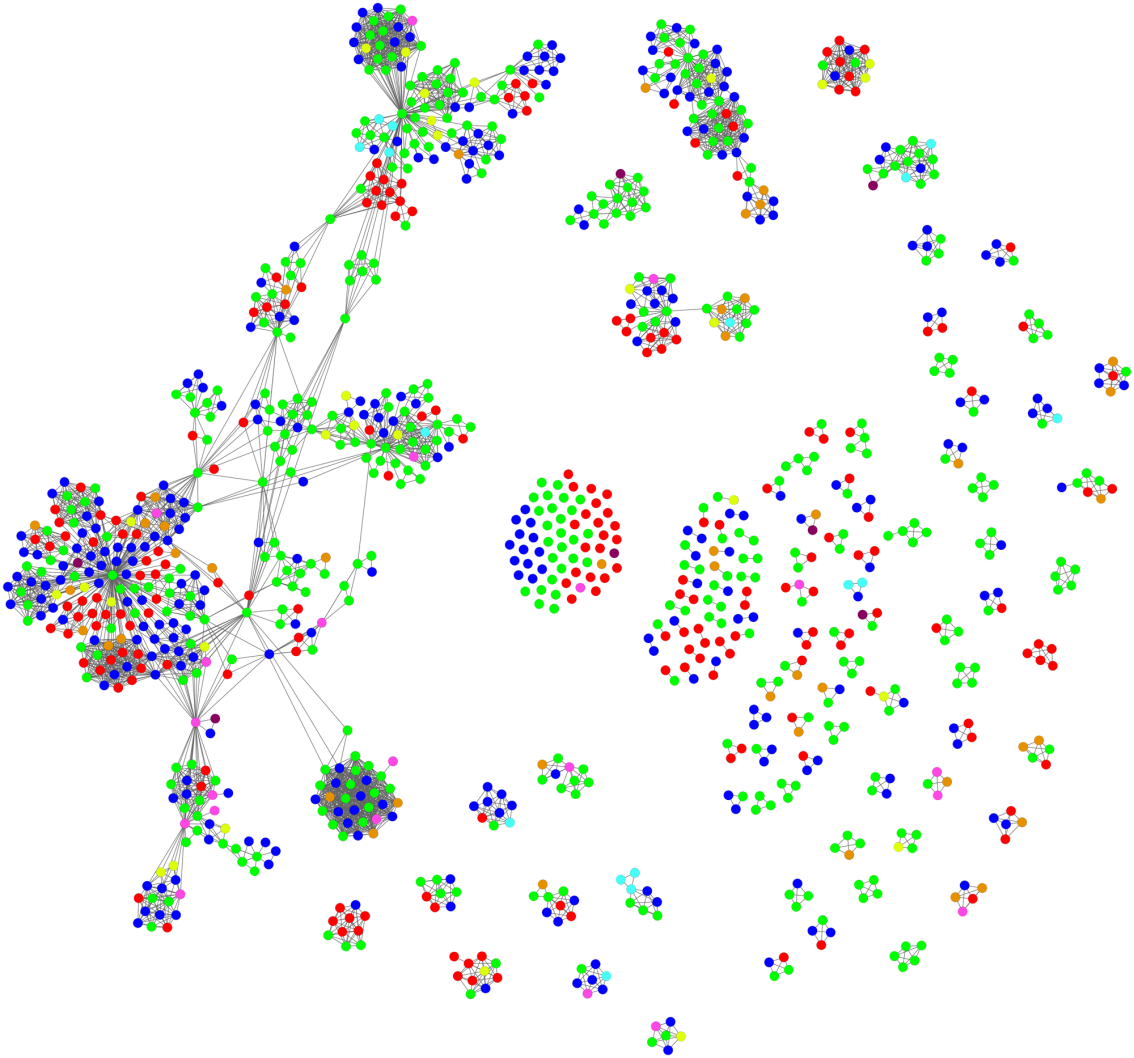
The network of CSOs (on the basis of conflicts). Note: The color-coding of CSOs is the same as that used in Table 1.

Given this context, the method used to depict the network of companies was used to illustrate the mining resistance network as well. Fig 2 illustrates this network, according to organization type. Here, colored nodes represent the different types of organizations involved in the resistance, as presented in Table 1.

## 4. Network effects in environmental justice

In this section, network effects in mining conflicts are examined. Different regression models were applied to the data to analytically uncover whether there are network effects in the formation of EJ outcomes. As previously noted, we investigate network effects in EJ outcomes by taking the network structure as given and analyzing the relation of the network structure to the EJ outcomes. Hence, what we do is not modelling or explaining the formation of the network. We rather use a given network formation for explaining the EJ scores in mining conflicts.

**Fig 3:**
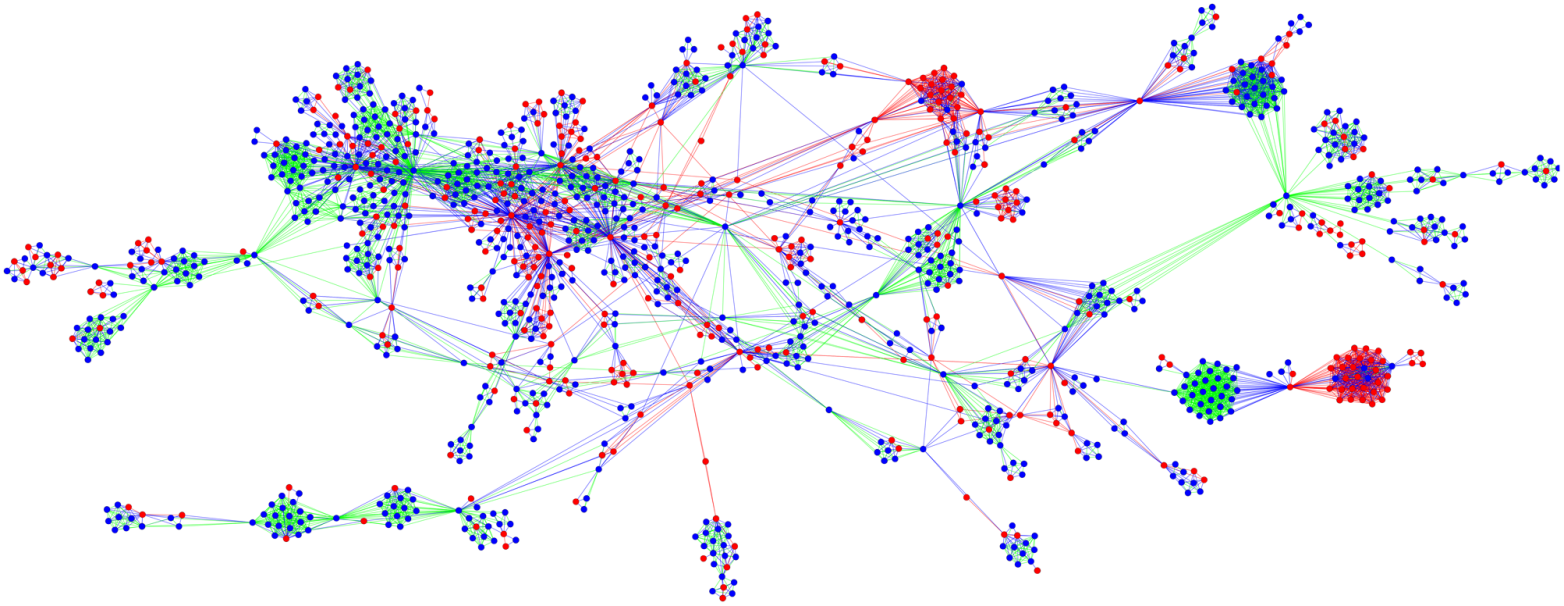
The GSCC network of CSOs and companies. Note: Red nodes and blue nodes represent companies and CSOs, respectively. If two companies are connected on the basis of a common conflict, the link is red. If a company and a CSO are connected through a common conflict, the links are shown in green and if two CSOs are connected on the basis of a common conflict, the links are shown in blue.

For the quantitative analysis, all CSOs and companies were first collected in a single network, where a CSO-CSO, company-company, or CSO-company pair was connected if both parties were involved in the same conflict (Fig 3 depicts the GSCC of this unified network). Here, the degree centrality of a CSO or a company indicates the number of other CSOs or companies it is connected to, while the PageRank centrality [62] of a CSO or a company indicates how important or central their connections are in the network (for details of the modified PageRank algorithm used in this paper, see [63]). Table 2 provides descriptive statistics for the unified network of CSOs and companies.

**Table 2:** Descriptive statistics for the unified network of CSOs and companies.

Indicator	CSOs	Companies
Number of nodes	1069	521
Average degree centrality	10.42	10.15
Max degree centrality	197	106
Average PageRank centrality	0.98	1.02
Max PageRank centrality	14.04	8.09

To investigate how EJ outcome of a conflict is shaped, a regression analysis using ordinary least squares and ordered probit models was applied to determine whether the number of companies and CSOs involved in a conflict and their average values for degree centrality and PageRank centrality played a role. Conflict characteristics were all controlled for including geographical location which refers to the continent where the conflict takes place, population type which reflects whether the conflict is in an urban or a rural area and income level of the host country. The results provided in Table 3 show that, in all models, only the number of CSOs in a conflict had a statistically significant impact on EJ scores. As the number of CSOs increased, the EJ score of a conflict was also more likely to be higher. However, this result was not dependent on the centrality of the participating CSOs or companies. According to these regressions, it was only the number of CSOs that had a significant impact, regardless of how central the participating CSOs and companies were.

**Table 3:** Determinants of the outcome of a conflict.

EJ score (on a scale of 0 to 5)	OLS	OLS	Ordered Probit	Ordered Probit
Number of companies	-0.03	-0.01	-0.02	-0.01
Degree centrality of companies	-0.01	-0.01	-0.01	-0.01
PageRank centrality of companies	0.15	-0.15	0.15	-0.14
Number of CSOs	0.12**	0.13**	0.08**	0.09**
Degree centrality of CSOs	-0.00	-0.01	0.01	0.00
PageRank centrality of CSOs	-0.22	-0.13	-0.23	-0.20
Geographical location dummies	No	Yes	No	Yes
Income dummies	No	Yes	No	Yes
Population dummies	No	Yes	No	Yes
# of observations	346	346	346	346
R-squared	0.08	0.18	0.02	0.07

** significant at a 1 percent level

Further analyses were conducted to determine whether the centrality of a CSO or a company in the network influenced the outcome of the conflict it was involved in. To this end, regression analysis was used to see whether the PageRank values of a CSO or company affected the average EJ score of the conflicts in which they took part. In this regression, the network topology was used to explain success in achieving EJ. In this regard, the regression model was similar to the one in Banerjee et al. [64] where the diffusion of information is explained by an individual’s eigenvector centrality in society. While the degree centrality of the CSO or company and the number of conflicts they were involved in were controlled for, dummy variables for CSO type and operation level (local, national or international) were included. Table 4 shows that the number of conflicts in which a CSO or company was involved had a negative impact on average EJ score. This is understandable and statistically expected: If a CSO or company participates in a greater number of conflicts, maintaining a relatively higher average score becomes more difficult. In addition, the PageRank of a CSO had a significant positive effect on the average EJ scores of the conflicts in which it was involved, while degree centrality had no significant effect. This shows that CSOs connected to central CSOs or companies in the network are more likely to achieve a higher EJ score. It must be noted that this result was controlled for degree centrality which indicates the size of a CSO. Hence, higher success was related to being more centrally located; not to other factors such as size. Given that this result was not due to the number of connections (degree centrality), it appears that being connected to central CSOs or companies in the network increases the likelihood of success for CSOs. The positive effect that PageRank has on EJ scores can be interpreted as the impact that the accumulated experiences of central CSOs have on environmental conflicts. This has strong policy implications: Connecting central CSOs to remote areas in the network—or to conflicts in which only minor CSOs participate—can help foster greater EJ.

**Table 4:** Determinants of the average outcomes for CSOs and companies.

Average EJ score of a CSO or a company	CSO network (OLS)	CSO network (OLS)	Company network (OLS)
**Number of conflicts**	-0.82**	-0.81**	-0.15
**Degree centrality**	0.00	0.01	0.01
**PageRank centrality**	1.49**	1.46**	0.18
**CSO dummies**	No	Yes	No
**Number of observations**	1069	1069	521
**R-squared**	0.06	0.08	0.01

** significant at a 1 percent level, no significance at a 5 percent level

**Fig 4:**
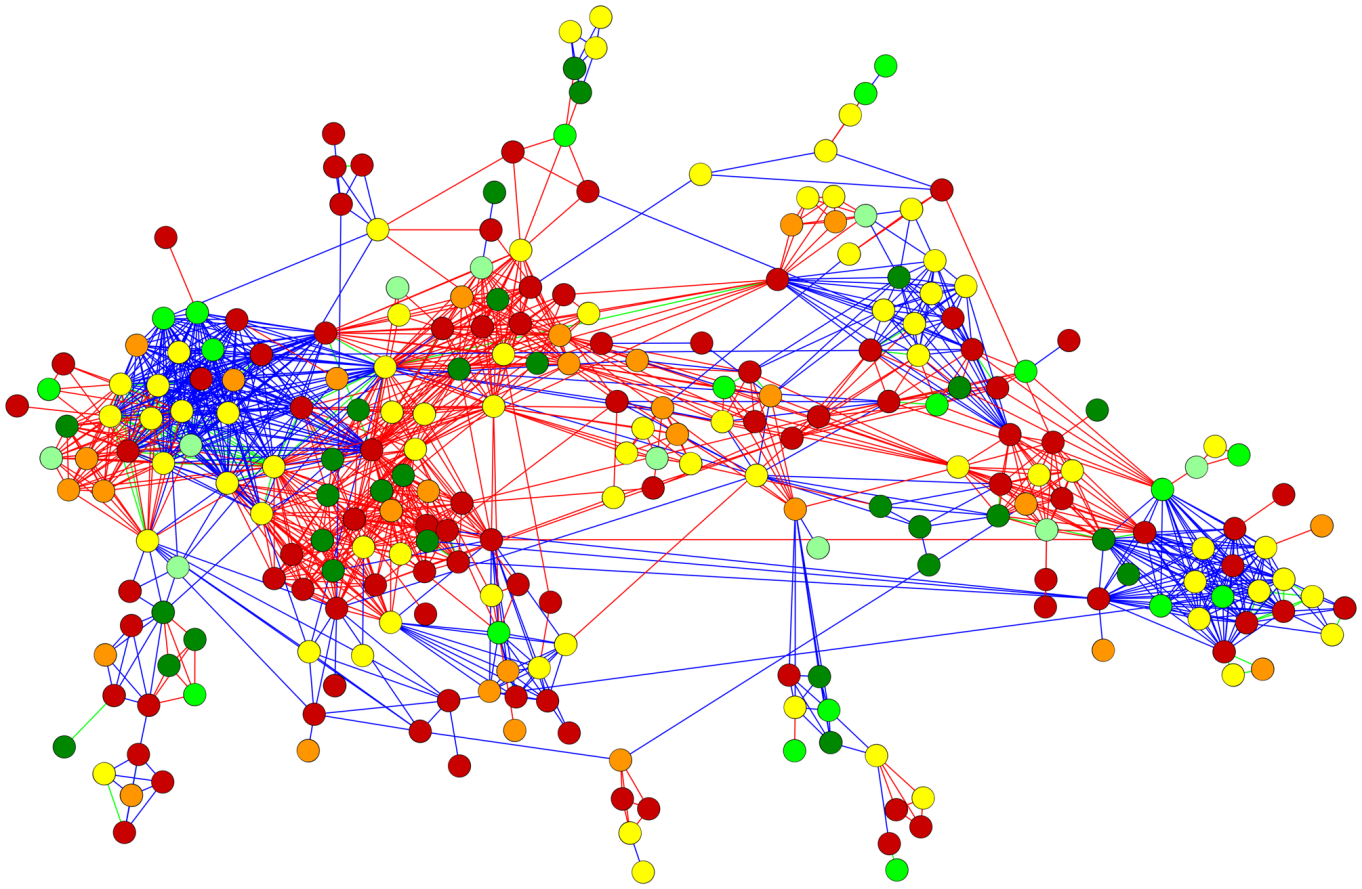
The GSCC of the network of environmental conflicts. Notes: Nodes depict conflicts and are color-coded on the basis of their EJ scores: Red nodes represent a score of 0, orange nodes 1, yellow nodes 2, light green nodes 3, green nodes 4, and dark green nodes 5. Conflicts linked through companies are shown in red, those linked through an CSO in blue, and if both CSOs and companies are involved they are linked in green.

One important aspect of a network is information flow and the way any given outcome spreads out through its connections. In our case, the question was whether connections in the network lead to similar outcomes for environmental conflicts. Fig 4 illustrates environmental conflicts in terms of their EJ scores. In this figure, if any CSO (or company) was involved in two conflicts, it was assumed that these conflicts were connected via the CSO (or company).

**Fig 5:**
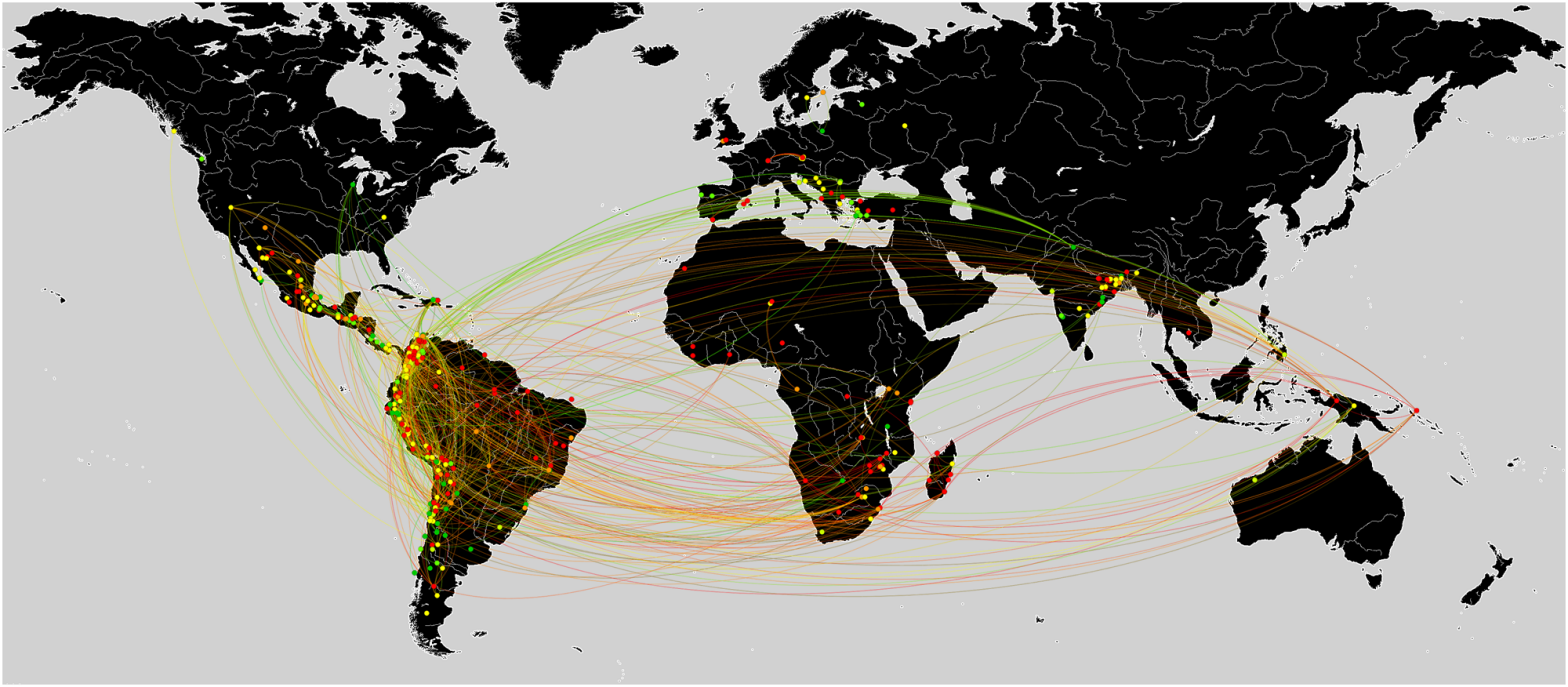
The network of environmental conflicts visualized on a world map.

An examination of the clusters in the network in Fig 4 reveals that conflicts that are in close proximity to one another tend to have similar EJ scores. This visual result hints at network effects in environmental conflicts. CSOs and companies pass on their experiences and know-how from one conflict to the next; hence, network distance between conflicts should matter. Another issue is whether network proximity is just a reflection of geographic proximity and whether conflicts have similar EJ scores due to similar geographical locations. Fig 5 depicts conflicts on a world map and clearly shows that geographical proximity alone cannot explain the variation in EJ scores. Conflicts in very similar locations can have very different EJ outcomes.

To show this empirically, regression analysis was applied to determine whether network distance had an effect on the EJ scores of two conflicts. To this end, the network of conflicts was defined through either the CSOs or companies, resulting in two types of distance between two conflicts: CSO-based distance, and company-based distance. CSO (company)-based distance denotes the length of the shortest path between two conflicts based on the links that CSOs (companies) form. For example, if the CSO (company)-based distance between two conflicts is one, then there is at least one CSO (company) common to both conflicts. If the CSO (company)-based distance between two conflicts is two, then there is no CSO (company) common to both conflicts but there is a third conflict where least one CSO (company) is common to these two conflicts.

The regressions presented here were exempt from the network autocorrelation problem [65] or in other words, the assumption of the independence of cases was not violated. This problem appears when the outcome variable is regressed on other outcome variables in the network, which in our case would correspond to regressing the EJ score of a conflict on scores of other conflicts. Network autocorrelation models are used in attempts to solve autocorrelation problem this type of regression Similarly, Exponential Random Graph Models (ERGM) are used to explain how a network forms [66]. Here, however, we had not set out to explain how the network of CSOs, companies or environmental conflicts were formed, thus, we did not regress the outcome of a conflict on the outcome of other conflicts. We were only interested in how the network distance between two conflicts impacted the similarity of their outcomes. Consequently, the independent variable, network distance, was exogenous to the dependent variable, similarity of outcomes. This shows that our model is not about network formation but rather explaining the similarity of EJ scores by a given network structure. This is actually why we used standard statistical techniques instead of models that focus on network autocorrelation or ERGM type of models.

We first regressed the absolute difference of EJ scores of two conflicts on network distance and controls (Table 5). Our aim was to investigate whether conflicts closer in terms of network distance had similar EJ scores. Regression results showed that when two conflicts were connected by CSOs, i.e. there was a CSO common to both conflicts, the absolute difference between their EJ scores decreased significantly, even if they were not connected through companies. This implies that conflicts connected by CSOs have similar EJ scores. In addition, in cases where company-based distance between two conflicts is one or two, the impact of shorter distance through CSOs increased. In short, the EJ scores of two conflicts were more likely to be similar, when they were both connected by CSOs and have a low distance by companies. This suggests that if two conflicts are connected only through companies and have no CSOs in common, the difference between their EJ scores does not decrease. All combinations of income levels, population types and geographical regions between the two conflicts were controlled for, indicating that these results are robust; this is also evident from the last two columns in Table 5, which reflect results from a regression on the conflict-fixed effects of one pivot conflict.

**Table 5:** Difference in EJ scores between two conflicts.

|ej1-ej2|	OLS	Ordered Probit	OLS	Ordered Probit	OLS	Ordered Probit	OLS	Ordered Probit
**cso1**	-0.16*	-0.11*	-0.15*	-0.11*	-0.14*	-0.10*	-0.10**	-0.08**
**cso2**	-0.07	-0.05**	-0.06	-0.04	-0.02	-0.01	-0.01	-0.01
**cso3**	0.01	0.00	0.01	0.01	0.05	0.03	0.02	0.02
**comp1**	0.04	0.03	0.04	0.03	0.07	0.04	0.04	0.03
**comp2**	0.10	0.06	0.10	0.06	0.14*	0.09*	0.09**	0.06**
**comp3**	0.03	0.03	0.04	0.03	0.07	0.04	0.06**	0.04**
cso1*comp1	-0.39*	-0.30*	-0.38*	-0.29*	-0.38*	-0.29*	-0.24	-0.22
cso1*comp2	-0.60**	-0.44**	-0.59**	-0.44**	-0.68**	-0.49**	-0.38**	-0.32**
cso1*comp3	-0.04	-0.05	-0.05	0.05	-0.12	0.00	0.11	0.16
cso2*comp1	-0.21	-0.19	-0.21	-0.19	-0.27	-0.22	-0.12	-0.15
cso2*comp2	-0.29	-0.19	-0.30	-0.19	-0.36*	-0.23	-0.24	-0.16
cso2*comp3	-0.23	-0.14	-0.24	-0.14	-0.29*	-0.18	-0.13	-0.07
cso3*comp1	-0.76**	-0.52*	-0.76**	-0.52*	-0.82**	-0.56*	-0.58*	-0.42
cso3*comp2	-0.08	-0.03	-0.08	-0.03	-0.11	-0.05	-0.02	0.01
cso3*comp3	0.08	0.06	0.08	0.06	0.03	-0.02	0.08	0.07
**same income type**			0.02	0.02				
**same population type**			-0.06	-0.04				
**same geography type**			-0.01	-0.00				
**Income controls**	No	No	No	No	Yes	Yes	Yes	Yes
**Geographical controls**	No	No	No	No	Yes	Yes	Yes	Yes
**Population controls**	No	No	No	No	Yes	Yes	Yes	Yes
**Conflict fixed effects**	No	No	No	No	No	No	Yes	Yes
**R-squared**	0	0	0	0	0.01	0	0.17	0.05
**# of observations**	77006	77006	77006	77006	77006	77006	77006	77006

** significant at a 1 percent level,

* significant at a 5 percent level

Note: For each conflict pair, one conflict was taken as the pivot conflict. Standard errors are clustered for pivot conflicts. “CSO i” (“comp i”) denotes the dummy variable for CSO (company)-based distance between two conflicts being “i”.

Similar regression analyses were run to test how network distance between two conflicts impacts the probability of having equal EJ scores. Table 6 shows that the likelihood of two conflicts having equal EJ scores is significantly higher when they are connected through CSOs and company-based distance between them is one or two. Hence, being closer in the network through CSOs and companies complementarily increases the likelihood of equal EJ scores.

**Table 6:** Probability that the EJ scores of two conflicts are equal.

(equal justice scores)	Probit	Probit	Probit	Probit
**cso1**	0.13**	0.13**	0.12*	0.10*
**cso2**	0.08	0.07	0.05	0.02
**cso3**	0.05	0.04	0.01	-0.01
**comp1**	-0.01	-0.01	-0.01	-0.00
**comp2**	0.01	0.01	0.01	-0.00
**comp3**	-0.05	-0.06*	-0.05	-0.04
cso1*comp1	0.33*	0.33*	0.31	0.25*
cso1*comp2	0.39*	0.39*	0.40**	0.29*
cso1*comp3	-0.52*	-0.51*	-0.52*	-0.68**
cso2*comp1	0.40	0.41	0.41	0.42
cso2*comp2	0.03	0.04	0.05	-0.03
cso2*comp3	0.11	0.11	0.11	-0.01
cso3*comp1	0.33	0.33	0.36	0.17
cso3*comp2	-0.13	-0.13	-0.14	-0.20
cso3*comp3	0.08	-0.08	-0.07	-0.12
**same income type**		-0.05**		
**same population type**		0.02		
**same geography type**		0.03		
**Income controls**	No	No	Yes	Yes
**Geographical controls**	No	No	Yes	Yes
**Population controls**	No	No	Yes	Yes
**Conflict fixed effects**	No	No	No	Yes
**R-squared**	0	0	0.01	0.08
**# of observations**	77006	77006	77000	77000

** significant at a 1 percent level,

* significant at a 5 percent level

Note: For each conflict pair, one conflict was taken as the pivot conflict. Standard errors are clustered for pivot conflicts.

The fact that distance between two conflicts has a significant impact shows that network effects in environmental conflicts go beyond direct connections. Network effects are not only due to a CSO or a company common to both conflicts, the proximity of two conflicts in the network also matters. Experiences, strategies, and the information that CSOs and companies spread throughout the network via their connections are all influential. If an environmental conflict with a high justice score is connected to others through CSOs and companies, the positive experiences will spread out through these connections. Based on the results provided in Table 2, it would also be better to connect conflicts that have high justice scores with other conflicts via central CSOs.

## 5. Synthesis and conclusions

The social network concept has become popular in the scholarly literature on social movements in general and on environmental justice in particular. This paper drew on the tools of social network analysis and built on a collaborative work that brought together quantitative information on mining conflicts, aiming to innovatively demonstrate that an EJ movement has indeed taken root in the form of a global social network in relation to mining conflicts, and that these networking efforts not only have a symbolic value in civil society, but also play an important role in EJ outcomes.

First, the analysis revealed that the greater the number of CSOs involved in a mining conflict, the more likely its outcome will be perceived as an EJ success. This coincides with empirical insights gained from case studies, which indicate that the size of a social movement actually matters in environmental resistance actions because large scale movements help achieve targeted objectives in several ways [52]. As Swain [67] notes, a social movement with massive support leads, above all, to the questioning of the legitimacy and representivity of the authorities and their policies. Obviously, this is not just about numbers, but about difference and plurality as well. Resistance movements that involve more CSOs are naturally more diverse, and given that EJ requires an understanding of the existence and importance of multiple perspectives, diversity—combined with mutual respect and solidarity—then serves to add strength to the movement. It incorporates “many different experiences people have in their environments; the cultures that informs those experiences and the various evaluations and reactions that emerge from them” [58, p. 125]. This finding also favors a network structure that is bottom-up, spontaneous and diverse over one single, centralized and formal organization. It indicates that grassroots movements may be successful in pursuing their own objectives, as indicated by Schlosberg [58]: There is power in grassroots movements, and hence, no particular need for institutionalized arrangements—more specifically, the organizing of the Big Ten.

Second, the analysis showed that if a CSO is connected to central CSOs, the average perception of EJ success is likely to increase. This means that in addition to finding strength and diversity in numbers, it is important for local resistance movements to develop the network at multiple scales because jumping scales and engaging with central national and international actors provides leverage for local activists. This result is very much in line with what Rootes [32,56] and Sikor and Newell [24] argue: In cases where national environmental organizations are resource-rich, linkages between local environmental campaigns and national and transnational organizations are extremely important and useful. In a number of well-known EJ successes, communities involved in strong networks were able to communicate to society the relevance of preventing mining exploration on the grounds of environmental, cultural, or legal values. Rivera [68], for instance, talks about how local level diversity in post-dictatorship Chile made access easier for international agencies and thus boosted visibility. Working with an array of national and international partners ultimately made it possible to share strategies and access independent assessments [69]; however, because bottom-up access from communities to national EJOs depends heavily on awareness levels in a given community, this is not always easy. Similarly, national EJOs may not always have the means to connect to international organizations. In such settings, professional groups (e.g., teachers, students, lawyers) may play key roles in helping local communities or national organizations raise awareness and carry the struggle onto a higher scale [56].

For local movements, the critical point is not to lose the grassroots while establishing links to higher levels. As Schlosberg [58] notes, when a movement is built, it starts from the bottom up, and there should be an awareness of the need to keep ownership in the hands of local participants. Moreover, networking at the national or international levels should not mean homogenizing local CSOs, but rather creating a platform for sharing relevant experiences and strategies that would strengthen them in their efforts to confront various issues. For example, innovative methods derived as a result of transnational networking might give social movements an initial strategic advantage vis-à-vis the national authorities.

Third, the paper shows that as network distance between two conflicts increases (or decreases), the more likely they are to lead to different (or similar) EJ outcomes. The key network effect here is the inverse relationship between the distance of two conflicts in the social network and the likelihood of sharing the same level of success. This result not only detects network effects in EJ movements for the first time within the context of mining conflicts, but also reinforces previous research that demonstrates these effects in other spheres, such as finance or in the context of economic crises.

What can we learn from this particular result? Can network analysis be used as an organization strategy to spread EJ success? While acknowledging diversity, difference and plurality, the results of this study expands on previous work by Schlosberg [58], who proposed that networks could be an alternative form of organizing that would remedy the limitations of the conventional model and free social movements from relying on more centralized organizations. The results of our analysis here offer insights into the kinds of bridges that can be built among diverse cases and the alliances that can be established across contexts and scales. Accordingly, it would be a strategic move on the part of successful CSOs to create links to other important conflicts and to disseminate information on how they achieved greater EJ success. Such insights underline the importance of highlighting achievements in EJ movements: publicizing and making the most of the successful cases so that they inspire other similar struggles. Overall, the message is clear: Network development should ultimately aim to achieve concrete gains that can be linked to the idea of success.

Naturally, building network links should not be read as establishing formal mechanisms among different organizations. On the contrary, as underlined again by Schlosberg [58] and Rootes [56], effective networking should go beyond organizational forms and be seen as mobile arrangements that welcome dynamic, heterogeneous and informal ties, that remain silent unless needed. This kind of organizing would minimize efforts required to maintain institutional support mechanisms, while maximizingresiliency and the ability to adapt to diverse and changing local conditions. It would include improving communication capacities and developing information strings to share experiences. Activist trust is undoubtedly another essential component of networking here [70].

In closing, the social network analysis presented in this paper is far from complete; it is based only on data reported by activists in relation to mining conflicts. Nonetheless, this visual representation exercise and discussion should be seen as a first step in showing the complex web of relationships among companies and resistance movements. From an EJ perspective, the commonalities in conflicts are so vast that the global village of social movements becomes quite small, and networks driven by decentralization, diversification and democratization seem to have the potential to create pathways that can change the power balance in favor of local communities.

## Supporting information

S1 File All relevant data used in the article. (XLSX)
